# Quantification of pancreatic proton density fat fraction in diabetic pigs using MR imaging and IDEAL-IQ sequence

**DOI:** 10.1186/s12880-019-0336-2

**Published:** 2019-05-14

**Authors:** Yidi Chen, Liling Long, Zijian Jiang, Ling Zhang, Delin Zhong, Xialing Huang

**Affiliations:** Radiology Department, The First Affiliated Hospital of Guangxi Medical University, No. 6 Shuangyong Road, Nanning, 530021 Guangxi China

**Keywords:** Magnetic resonance imaging, Pancreas, Visceral steatosis, Diabetes mellitus, Animal experiments

## Abstract

**Background:**

Recent studies have highlighted the correlation between diabetes and pancreatic fat infiltration. Notably, pancreatic fat content (PFC) is a potential biomarker in diabetic patients, and magnetic resonance imaging (MRI) provides an effective method for noninvasive assessment of pancreatic fat infiltration. However, most reports of quantitative measurement of pancreatic fat have lacked comparisons of pathology results. The primary objective of this study was to determine the feasibility and accuracy of pancreatic MRI by using pancreatic fat fraction (PFF) measurements with the IDEAL-IQ sequence; the secondary objective was to explore changes in PFC between pigs with and without diabetes.

**Methods:**

In this prospective study, 13 Bama Mini-pigs (7 females, 6 males; median age, 2 weeks) were randomly assigned to diabetes (*n* = 7) or control (*n* = 6) groups. Pigs in the diabetes group received high fat/high sugar feed, combined with streptozotocin injections. At the end of 15 months, biochemical changes were evaluated. All pigs underwent axial MRI with the IDEAL-IQ sequence to measure PFF; PFC of fresh pancreatic parenchyma was measured by the Soxhlet extraction method; and pancreatic fat distribution was observed by histopathology. Results of all analyses were compared between the diabetes and control groups by using the Mann-Whitney U-test. Correlations of PFF and PFC, fasting blood glucose (GLU), and serum insulin (INS) were calculated by using the Spearman correlation coefficient. Single-measure intraclass correlation coefficient (ICC) was used to assess interreader agreement.

**Results:**

There were significant differences between diabetes and control groups: GLU (mmol/L) was 18.06 ± 6.03 and 5.06 ± 1.41 (*P* < 0.001); INS (mU/L) was 21.59 ± 2.93 and 29.32 ± 3.27 (*P* = 0.003); PFC (%) was 34.60 ± 3.52 and 28.63 ± 3.25 (*P* = 0.027); and PFF (%) was 36.51 ± 4.07 and 27.75 ± 3.73 (*P* = 0.003). There was a strongly positive correlation between PFF and PFC (r = 0.934, P < 0.001); there were moderate correlations between PFF and GLU (r = 0.736, *P* = 0.004; positive correlation), and between PFF and INS (r = − 0.747, P = 0.003; negative correlation). Excellent interreader agreement was observed for PFF measurements (ICC, 0.954).

**Conclusions:**

Pancreatic fat infiltration shows a clear association with diabetes. MRI with the IDEAL-IQ sequence can be used to accurately and reproducibly quantify PFC.

## Background

Pancreatic fat infiltration may be associated with several diseases involving the pancreas, including type 2 diabetes, pancreatitis, pancreatic cancer, cystic fibrosis, haemochromatosis [1, 2], and vertebral body density fat fraction [3]. The role of pancreatic fat infiltration in the pathogenesis of type 2 diabetes has been increasingly emphasized, such that the relationship between pancreatic fat content (PFC) and type 2 diabetes has attracted greater research interest [4–6]. Therefore, there is a growing demand for assessment of adipose tissue [7, 8], including accurate quantification of pancreatic fat fraction (PFF) via magnetic resonance imaging (MRI) [9, 10].

There are multiple medical imaging technologies to evaluate and quantify pancreatic fat infiltration in patients with obesity or diabetes; these include abdominal ultrasound [11], computed tomography [12], and MRI [13]. Currently, noninvasive quantitative MRI pulse sequences enable measurement of PFF [14]. The iterative decomposition of water and fat with echo asymmetry and least-squares estimation quantitation (IDEAL-IQ) sequence uses a multi-echo water lipid separation technique, which can eliminate the interference of tissue T_2_* in the process of fat quantification, by using a multi-echo signal variation curve; this enables accurate quantification of visceral fat [15].

Understanding the precision of IDEAL-IQ measurements with MRI is critical for establishing PFF as a noninvasive parameter for detection, quantification, and monitoring of pancreatic steatosis. However, most previous reports have comprised clinical research; the investigators determined pancreatic fat deposition in non-alcoholic steatohepatitis patients [16] and discussed the correlation between PFC and fasting blood glucose (GLU) in patients with type 2 diabetes [17]; however, those studies did not involve confirmation of the accuracy of IDEAL-IQ measurements from pathology analyses. In addition, there are differing perspectives regarding whether PFC is related to prediabetes or diabetes [18].

To resolve these problems, appropriate animal models are needed. Notably, the Bama Mini-pig shows similarity with regard to the functions of human organs, as well as the morphological structure of the pancreas and its physiological characteristics [19]. Bama Mini-pigs and humans are both omnivorous animals; thus, they exhibit similar sugar and lipid metabolism. Moreover, the Bama Mini-pig has been widely applied in studies of atherosclerosis and human metabolic diseases, such as diabetes. The design of diabetes models in Bama Mini-pigs is relatively mature in our institution; thus, this experiment uses the Bama Mini-pig as the experimental model [20].

The purpose of this study was to assess the accuracy of the IDEAL-IQ sequence for quantifying PFF in an animal model using pathology analyses for confirmation, as well as to explore changes in PFC in animals with or without diabetes. Therefore, we established an animal model for scanning by the IDEAL-IQ sequence, and acquired fresh pancreas specimens; we then performed pathology analysis and determined PFC.

## Methods

### Diabetic animal models

Experimental animals were acquired from the School of Animal Science and Technology at Guangxi University (closed colony animals; batch number: SCXK (GUI) 2013–0003). Our prospective study was approved by the ethics committee of our institution, approval number: 2018 (KY-E-011). The care of laboratory animals and all animal experiments adhered to the Guide for the Care and Use of Laboratory Animals published by the United States National Institutes of Health. At the end of the experiment, all pigs were euthanized by using 100 mg/kg pentobarbital.

Thirteen young Bama Mini-pigs (7 females, 6 males; median age, 2 weeks) were randomly assigned to diabetes (*n* = 7; 4 females, 3 males) or control (*n* = 6; 3 females, 3 males) groups by using a random number table. The standard laboratory pellet diet was used until the end of the 5th month; beginning in the 6th month, the diabetes group received a high-fat, high-sugar diet (standard diet with 37% sucrose and 10% fat). In the 8th month, small doses of streptozotocin (50 mg/kg body weight; Sigma-Aldrich Chemical, St. Louis, MO, USA) were injected 3 times consecutively at intervals of 1 week. The streptozotocin was dissolved in sterile citric acid–sodium citrate buffer solution to a concentration of 5%; 50-ml syringes were used to immediately administer this prepared solution intravenously via the marginal ear vein over a period of 5 min. In contrast, the control group received the standard laboratory pellet diet for this period; these animals were administered identical volumes of citric acid–sodium citrate buffer solution (without streptozotocin) at the time of streptozotocin injection to animals in the diabetes group. The animals were kept in similar cages with a 12-h light-dark cycle, as well as constant temperature and humidity. They had free access to water.

In our study, we used the 2018 American Diabetes Association diagnostic criteria for human diabetes [21]; when GLU levels were greater than 7.0 mmol/L or random blood glucose levels were greater than 11.0 mmol/L, we defined the diabetic animal model to be successful.

Gravimetry and metabolic profiles of all enrolled pigs were obtained during a 15-month acclimation period. Venous blood (approximately 10 ml) was extracted from those pigs with empty stomachs and sent to the clinical laboratory of our institution for measurement of GLU; serum insulin (INS), total cholesterol (TCH), triglycerides (TG), low-density lipoprotein (LDL), high-density lipoprotein (HDL), and other metabolic profiles were determined at the end of the acclimation period. These tests were performed by automatic chemiluminescence immunoassay analysis; all detection kits were provided by Shanghai Zhicheng Biological Technology Co., Ltd.

### MRI examination and IDEAL-IQ MR sequence

After the diabetes model was successfully established (15 months of age), pancreatic MRI scans were performed by a 3.0-T imager (GE Discovery 750 Plus; GE Healthcare; Little Chalfont, UK) with an eight-channel body phase array extremity coil. MR imaging was accelerated by using array spatial and sensitivity encoding. Routine MR imaging included axial gradient echo sequence T1-weighted imaging (T1WI) (TR 3.7 ms, TE 2.0 ms, flip angle 12°, section thickness 4.0 mm, layer spacing 1.5 mm, FOV 420 mm × 420 mm, matrix 256 × 256), coronal half Fourier single excitation fast spin echo sequence T2-weighted imaging (T2WI) (TR 1800.0 ms, TE 90.0 ms, flip angle 180°, thick 4.0 mm, layer spacing 2.0 mm, FOV 420 mm × 420 mm, and matrix 384 × 256), and fat suppression T2WI sequence (TR 3000.0 ms, TE 85.0 ms, flip angle 110°, thickness 4.0 mm, layer spacing 1.0 mm, FOV 420 mm × 420 mm, and matrix 320 × 320).

IDEAL-IQ imaging was performed by collecting 6 echo signals and estimating complex domain mapping by the iterative least-squares method. Complex domain reconstruction was used to distinguish water from fat and obtain a dynamic 0–100% fat ratio. Imaging parameters of the axial IDEAL-IQ sequence were as follows: TR: 5.9 ms, minimum TE 1.3 ms, flip angle 3°, echo train length 3, section thickness 4.0 mm, layer spacing 0.15 mm, FOV 420 mm × 420 mm, matrix 160 × 160, pixel size 19.3 mm, and NEX 2 times.

The IDEAL-IQ images were analyzed by using an imaging workstation (Advantage Workstation 4.6; GE Healthcare). The PFF was measured by Fat Fraction Mapping, which was obtained from the IDEAL-IQ sequence. Region of interest (ROI) measurements included the periductal and marginal parenchyma of the pancreas, with approximately 1.5–2.0 cm in diameter, but were careful not to include the surrounding adipose tissue. In our study, we placed ROIs in the same pancreatic regions in all pigs, such that the ROIs included the pancreatic head, body, and tail. The pancreatic head, body, and tail were measured three times with ROIs of similar size, and then average amounts were calculated. Two blinded examiners (radiologists) independently measured IDEAL-IQ images; both examiners had 5 years of experience in abdominal MR imaging.

### Histologic analysis and fresh pancreatic fat measurement

On the same day after the MRI scan was completed, pigs were weighed and euthanized by using 100 mg/kg pentobarbital. Samples of the pancreas were obtained; then, six pieces of tissue in the pancreatic head, body, and tail were cut (volumes of 0.5–1.0 cm^3^). These cut pieces of tissues were fixed in 4% polyformaldehyde for 24 h, then processed by using a tissue processor and embedded in paraffin. Multiple 5-μm-thick slices were obtained, then stained with hematoxylin and eosin to estimate the extent of pancreatic steatosis. Selected tissue samples from the same region were used to obtain frozen sections, which were stained with Oil Red to estimate the pancreatic fat distribution. All histologic analyses were performed by a pathologist with 10 years of experience in diagnosis of abdominal pathology, from the Pathology Department of our institution; the pathologist was blinded to the results of MRI analysis.

All remaining pancreatic tissues from the pathology samples were sent to the Guangxi Region Center for Analysis and Test Research (Registration No. CNAS L1571), and fresh PFC was obtained by the Soxhlet extraction method [22]. The formula for the content of fat in the sample is: X = (M1- M0) / M2 × 100%. In this formula, X is the fat content of the test specimen, in g/100 g; M1 is the weight of the receiving bottle and fat after constant weight, in g; M0 is the weight of the receiving bottle, in g; M2 is the total mass of the test specimen, in g; and 100% is the conversion factor.

### Statistical analyses

Consistent PFF measurements between two observers were assessed with the Bland-Altman method (version 16.4.3; MedCalc Software, Ostend, Belgium). The single-measure intraclass correlation coefficient (ICC) was used to assess interreader agreement. The following guidelines were used for interpretation of kappa or ICC inter-rater agreement measures [23]: less than 0.40, poor; between 0.40 and 0.59, fair; between 0.60 and 0.74, good; between 0.75 and 1.00, excellent.

All statistical analyses were performed by using SPSS 22.0 Windows Student Version statistics software (IBM, Armonk, NY, USA). All variables are expressed as mean ± standard deviation ($\overline{x}\pm s$). The Kolmogorov-Smirnov method was used to test whether the data exhibited a normal distribution; the Mann-Whitney U-test was used to judge differences in weight, PFC, PFF, GLU, and INS between diabetes and control groups. One-way analysis of variance (ANOVA) was used to compare fat content in pancreatic head, body, and tail. The correlations of PFF with PFC, GUL, and INS were calculated by using the Spearman correlation coefficient. Statistical significance was set at *P* < 0.05.

## Results

At the fifteenth month, when the diabetes model was successfully implemented, diabetic pigs (7/13) exhibited significant increases in weight, GLU, TCH, TG, and LDL; concomitantly, they showed significant reductions in INS and HDL (Table 1).

**Table 1:** Comparison of clinical biochemistry characteristics between diabetic and control pigs

Variable	Diabetic pigs (*n* = 7)	Control pigs (*n* = 6)	*p* value
Age (months)	14.72 ± 2.21	15.17 ± 1.47	0.303
Weight (kg)	88.99 ± 11.85	58.67 ± 9.22	<0.001
Gender	*F* = 4, M = 3	*F* = 3, M = 3	0.805
Diabetic status			
GLU (mmol/L)	18.06 ± 6.03	5.06 ± 1.41	<0.001
INS(mU/L)	21.59 ± 2.93	29.32 ± 3.27	0.003
Lipid status			
TCHO (mmol/L)	4.36 ± 1.14	2.98 ± 0.56	0.021
LDL (mmol/L)	2.59 ± 0.65	1.89 ± 0.48	0.015
HDL (mmol/L)	1.24 ± 0.20	1.69 ± 0.29	0.007
TG (mmol/L)	3.63 ± 1.64	0.89 ± 0.38	0.002

Note: *F* Female, *M* Male, *GLU* Fasting blood glucose, *INS* Serum insulin, *TCHO* Total cholesterol, *TG* Triglycerides, *LDL* Low-density lipoprotein, *HDL* High-density lipoprotein

**Fig. 1:**
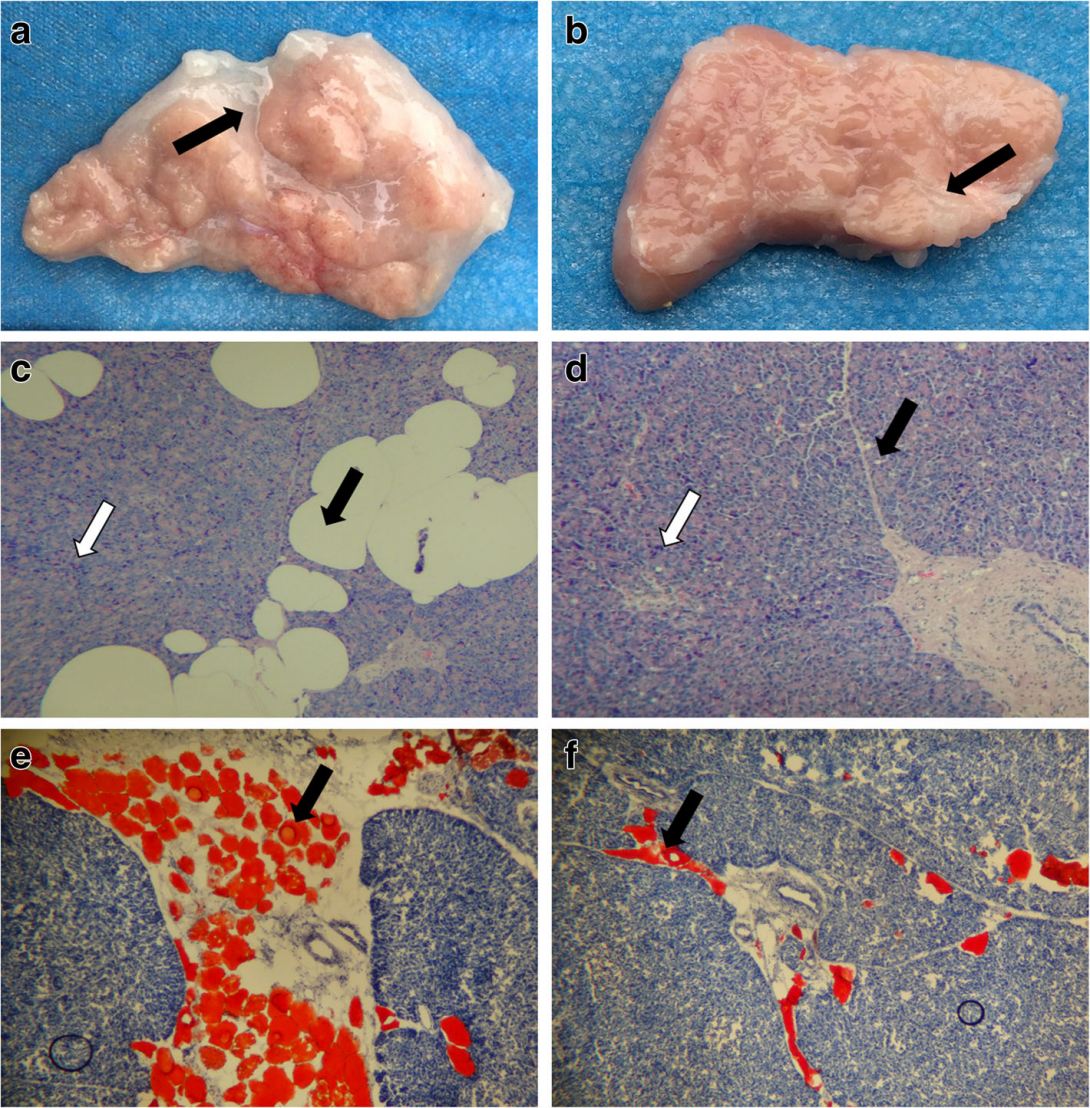
Histopathological features of the pancreas. (**a**, **b**) Transverse histologic sections of the pancreas, the adipose tissue (black arrow) in the diabetic group (**a**) was increased, compared with the control group (**b**). (**c**, **d**) Hematoxylin-eosin stain (original magnification × 100), the number of fat cells (black arrow) in the diabetic group (**c**) was increased, while the number of pancreatic islet cells (white arrow) was reduced, compared with the control group (**d**). (**e**, **f**) Oil Red O stain (original magnification × 100), adipose tissue appears orange, the degree of fat infiltration. (black arrow) in the diabetic group (**e**) was increased, compared with the control group (**f**)

Pancreatic gross pathology specimens showed a reddish color in the diabetes group. The whole pancreas exhibited reduced parenchymal attenuation; pancreatic fatty infiltration was increased with an uneven distribution, such that fat was observed more frequently in pancreatic circumjacent areas in the diabetes group (Fig. 1a, b). Hematoxylin-eosin staining showed that the range of steatosis was higher in diabetic pigs than in control pigs, while the number of pancreatic parenchymal cells was lower in diabetic pigs than in control pigs (Fig. 1c, d). Oil Red staining showed that the fat distribution was heterogeneous in the central and peripheral zones of the pancreas, and the extent of fat infiltration in the central region of the pancreas was markedly increased in diabetic pigs, compared with control pigs (Fig. 1e, f).

The PFC and pancreatic PFF in the diabetes group were higher than those in control group; PFC (%) was 34.60 ± 3.52 and 28.63 ± 3.25 (*P* = 0.027), and PFF (%) was 36.51 ± 4.07 and 27.75 ± 3.73 (*P* = 0.003), respectively (Table 2).

**Table 2:** The PFC and PFF of the Bama pigs on the fifteenth month

Variable	Diabetic pigs	Control pigs	*Z* Value	*P* Value
PFC (%)	34.60 ± 3.52	28.63 ± 3.25	−2.22	0.027
PFF (%)	36.51 ± 4.07	27.75 ± 3.73	−3.00	0.003
*n*	7	6	–	–

Note: *PFC* Fresh pancreatic fat content, *PFF* Pancreatic fat fraction

**Fig. 2:**
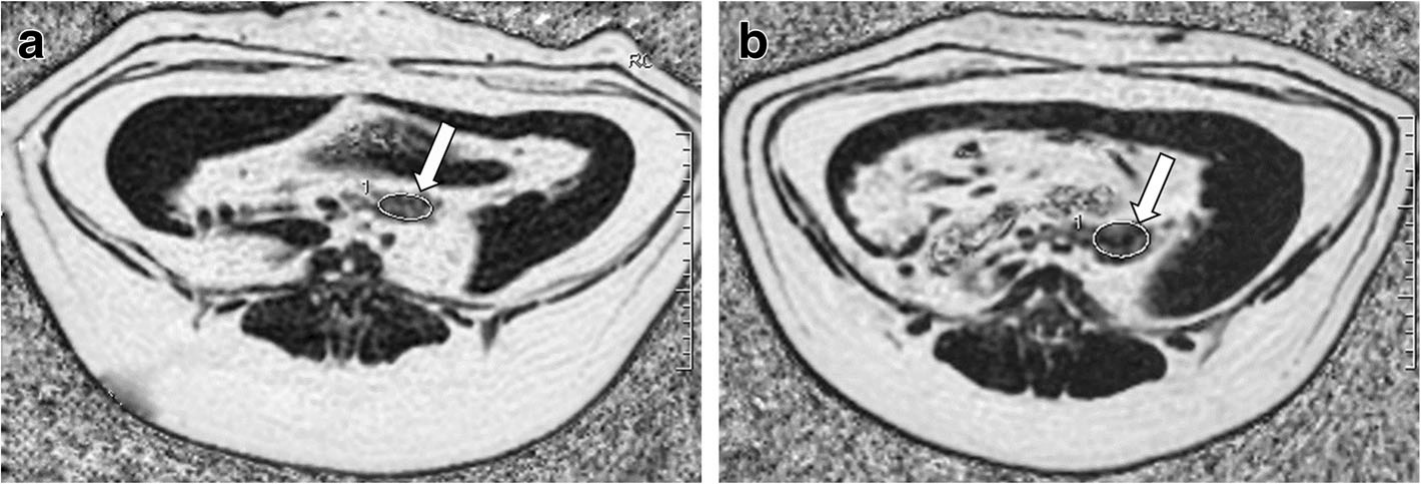
Pancreatic fat fraction (PFF) mapping by the IDEAL-IQ sequence. The PFFs were 34.70% in the diabetes group (**a**) and 26.93% in the control group (**b**), respectively (white arrows)

There were no significant differences in the PFF of the pancreatic head, body, and tail, as measured by MRI. PFF measurements of the pancreatic head, body part, and tail of the diabetic group were 34.82 ± 3.89%, 36.51 ± 4.07%, and 35.89 ± 4.91% (*P* = 0.762). In the control group, these were 28.96 ± 3.55%, 27.64 ± 3.81%, and 28.34 ± 3.12% (*P* = 0.809) (Table 3, Fig. 2).

**Table 3:** ANOVA of the PFF in pancreatic head, body and tail

	*n*	pancreatic head	pancreatic body	pancreatic tail	F	*P* Value
Diabetes	7	34.82 ± 3.89(%)	36.51 ± 4.07(%)	35.89 ± 4.91(%)	0.276	0.762
Control	6	28.96 ± 3.55(%)	27.64 ± 3.81(%)	28.34 ± 3.12(%)	0.215	0.809

Note: *ANOVA* One-way analysis of variance

**Fig. 3:**
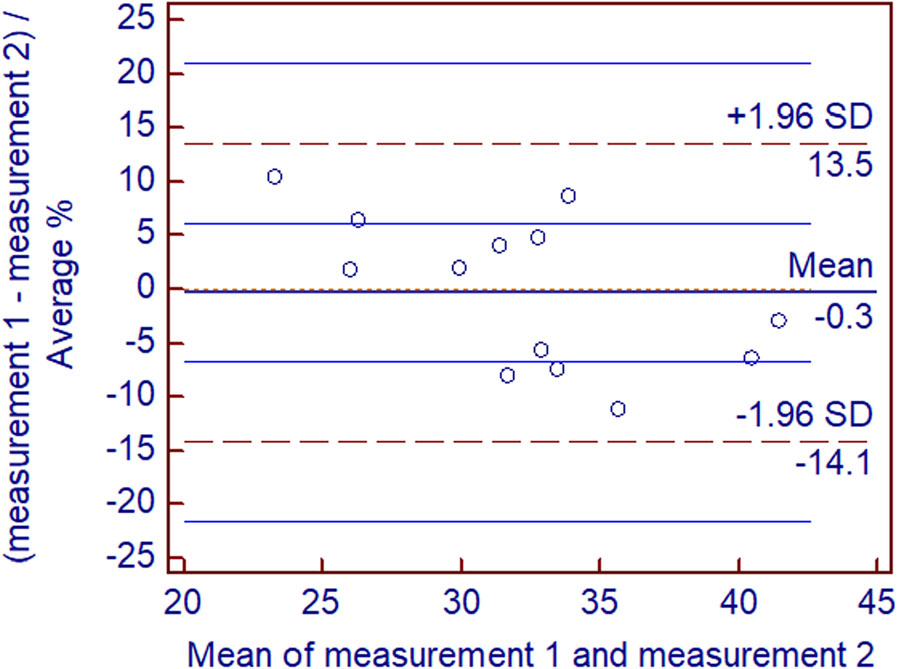
Bland-Altman difference plots for PFF measurements generated by two different readers blinded to each other. Dotted red lines demarcate 1.96 standard deviations (SD), and blue lines depict their 95% prediction limits

The interclass correlation coefficient (ICC) in the two measurements was 0.954 (95% confidence interval [CI]: 0.848, 0.986; *P* < 0.001); Bland-Altman plots showed good interobserver agreement of PFF measurements between the two observers (Fig. 3).

**Fig. 4:**
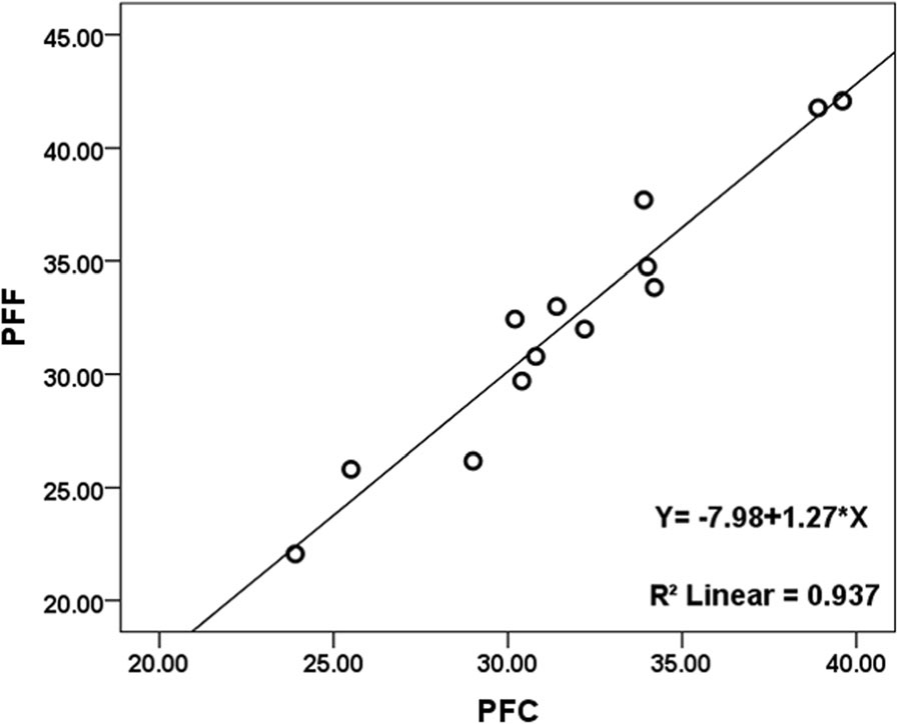
Relationship between PFC and PFF in the thirteen pigs. As the pancreatic fat content increases, the pancreatic fat fraction following increased which measured by MRI with IDEAL-IQ sequence, there is a very strong linear correlation coefficient between PFC and PFF

**Fig. 5:**
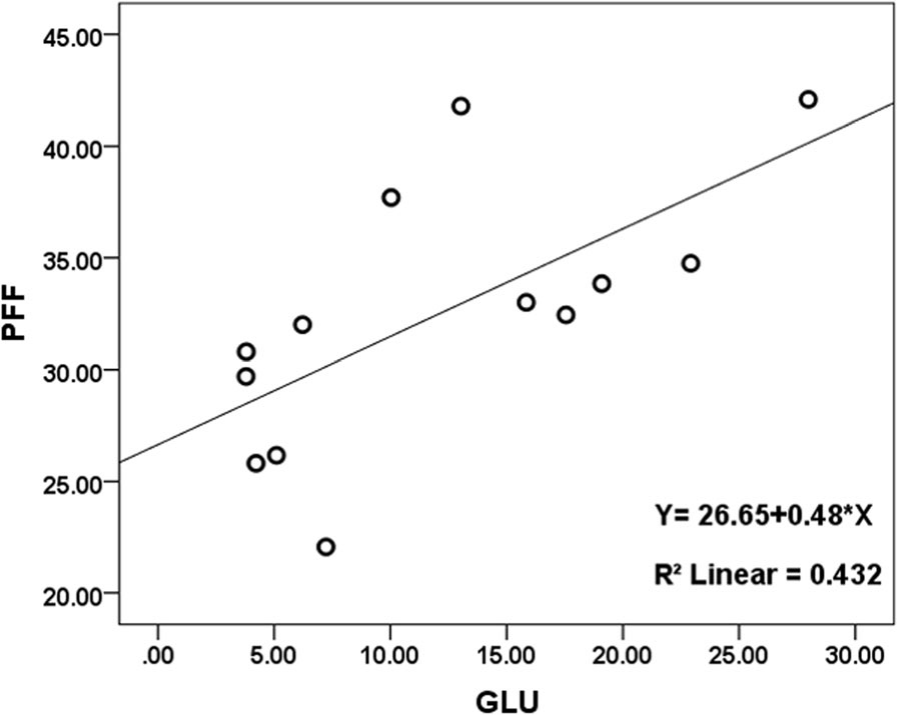
Relationship between PFF and GLU (fasting blood glucose) in the thirteen pigs. As the fasting blood glucose increases, the pancreatic fat fraction following increased, there is a moderate positive correlation between PFF and GLU

**Fig. 6:**
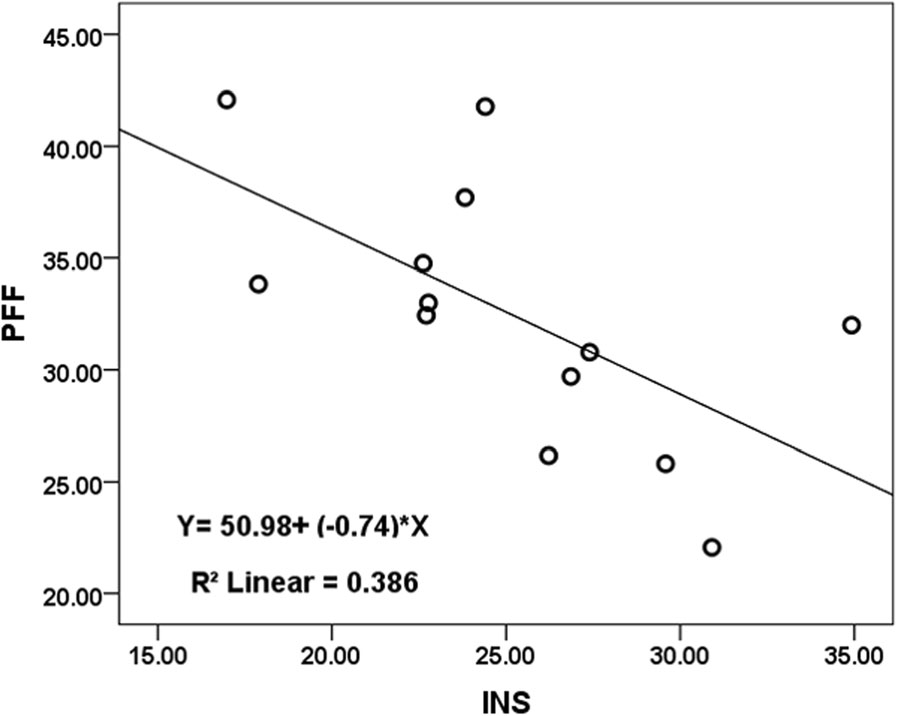
Relationship between PFF and INS (serum insulin) in the thirteen pigs. As the pancreatic fat fraction increases, the serum insulin following reduced, there is a moderate negative correlation between PFF and INS

There was a strongly positive correlation between PFF and PFC (r = 0.934; *P* < 0.001; 95% CI: 0.714, 0.999), and there were moderate correlations between PFF and GLU (*r* = 0.736; *P* = 0.004; 95% CI: 0.313, 0.916; positive correlation), as well as between PFF and INS (*r* = − 0.747; *P* = 0.003; 95% CI: -0.933, − 0.306; negative correlation) (Table 4, Figs. 4, 5, 6).

**Table 4:** Correlation analysis between PFF with PFC, GLU and INS

	*n*	*r*	*P* Value	95% CI
PFC	13	0.934	<0.001	0.714~0.999
GLU	13	0.736	0.004	0.313~0.916
INS	13	- 0.747	0.003	- 0.933~ − 0.306

Note: *GLU* fasting blood glucose, *INS* Serum insulin, *PFC* Fresh pancreatic fat content, *PFF* Pancreatic fat fraction

## Discussion

In this animal model-based study, we investigated the relationships among PFF (measured by using MRI with IDEAL-IQ sequence), PFC, and a variety of risk factors associated with diabetes mellitus. Prior studies have shown that biopsy is difficult and many complications are involved in assessment of diabetic pancreatic pathology; such studies often involve the use of human organ donor tissue samples [24, 25] or the establishment of animal models [26, 27]. Hu et al. found that the IDEAL IQ sequence can be used for quantitative evaluation of vertebral fat deposition in alloxan-induced diabetic rabbits; however, changes in PFC remain unclear in the context of diabetic rabbits [28]. Importantly, unlike previous MRI techniques used to evaluate visceral fat, the IDEAL-IQ enables adjustment for common biases in the measurement of tissue fat, including T_1_ and T_2_* effects, as well as main magnetic field inhomogeneity [29, 30].

To establish a model of diabetes with metabolic characteristics similar to those of humans, and to obtain pancreatic specimens to study changes in fat content and fat deposition, Bama Mini-pigs were used in the present study. As previously noted, pigs and humans exhibit sugar and lipid metabolism are very similar, such that the Bama Mini-pig has been widely applied in studies of atherosclerosis and human metabolic diseases, such as diabetes research [22, 31]. In the pre-experiment, we founded that the pig’s pancreas was more likely to be observed in the MR imaging and the shape of the porcine pancreas was similar to that of humans, whereas the shape of the pancreas in rabbits and rodents was not similar. Thus, our study used Bama Mini-pigs as the experimental model.

Our study used fat fraction mapping, obtained by using the IDEAL-IQ sequence, to measure PFF. Linear correlation analysis of all 13 pigs revealed that the PFC and PFF exhibit a very strong positive correlation (r = 0.934, *P* < 0.001). Notably, the PFC was measured by the Soxhlet extraction method, which enables determination of the true fat content of pancreatic tissue. Therefore, we speculate that PFF could accurately reflect the fat content of the pancreas. To verify consistency among multiple PFF measurements, the two observers in this study independently measured the fat components of the pancreas. The ICC of the two measurements was 0.954 (95% CI: 0.848, 0.986; P < 0.001); Bland-Altman plots showed good interobserver agreement of PFF measurements. Furthermore, our results showed that the reproducibility of PFF measurements was similar to that of PFC measurements performed by ^1^H magnetic resonance spectroscopy [32].

Increasing evidence suggests that individuals with excessive fat in the pancreas are at greater risk of chronic metabolic disorders; fatty pancreas disease is a frequent clinical entity, associated with a markedly increased risk of metabolic syndrome and its components (e.g., diabetes mellitus) [33]. Pancreatic fat infiltration is related to obesity and has important clinical significance in terms of glucose metabolism; further, it is associated with an increased prevalence of type 2 diabetes [34, 35]. Singh et al. reported that circulating levels of triglycerides and glycated hemoglobin may serve as markers for pancreatic fat; however, the noninvasive and accurate detection of pancreatic fat needs further exploration [36]. Our results showed that there were moderate correlations between PFF and GLU (*r* = 0.736, *P* = 0.004), as well as between PFF and INS (*r* = − 0.747, *P* = 0.003); these indicate that the degree of fat infiltration in the pancreas is related to changes in GLU and INS.

In this study, pathology analysis revealed that pancreatic fat deposition comprises inhomogeneity between the central region near the main pancreatic duct and the lobulated region near the margin; therefore, it was difficult to obtain objective and accurate PFC measurements by tissue biopsy. However, our results demonstrate that MRI scanning with the IDEAL-IQ sequence for pancreatic fat quantitation could obtain relatively stable and accurate PFF results; thus, PFF may serve as a biomarker to predict the severity of pancreatic lesions during the development of diabetes in humans.

Nonetheless, our study has a few limitations. First, our study included only 13 Bama Mini-pigs and was therefore relatively small. However, our study included an adequate sample size to detect clinically meaningful correlations, as shown by statistical analysis. Second, although we assessed the repeatability of PFF measurements for different observers, we did not assess this repeatability for multiple imaging platforms. Finally, we did not include blood lipids, or other metabolic markers, and we did not analyze their correlations with pancreatic fat infiltration; this will be performed in our subsequent studies.

## Conclusion

Our study revealed greater pancreatic fat infiltration in diabetic pigs than in control pigs; moreover, PFC was well-correlated with GLU in this model. This study also verified that the IDEAL-IQ sequence of MRI can accurately and reproducibly measure PFF.

## Abbreviations

CI: confidence interval
GLU: fasting blood glucose
HDL: high-density lipoprotein
ICC: intraclass correlation coefficient
IDEAL IQ,: iterative decomposition of water and fat with echo asymmetry and least-squares estimation quantitation
INS: serum insulin
LDL: low-density lipoprotein
PFC: pancreatic fat content
PFF: pancreatic fat fraction
ROI: region of interest
TCH: total cholesterol
TG: triglycerides
